# Disease profile in a cohort of Brazilian patients diagnosed with alpha-mannosidosis

**DOI:** 10.1016/j.ymgmr.2025.101220

**Published:** 2025-04-11

**Authors:** Fabiano de Oliveira Poswar, Tamires Silva Alves, Daniel Rocha de Carvalho, Hélio van der Linden, Charles Marques Lourenço, Dafne Dain Gandelman Horovitz, Anneliese Barth, Carmen Silvia Curiati Mendes, Ana Maria Martins, Roberto Giugliani

**Affiliations:** a Medical Genetics Service, HCPA, Porto Alegre, Brazil; b Postgraduate Program in Genetics and Molecular Biology, UFRGS, Porto Alegre, Brazil; c Postgraduate Program in Child and Adolescent Health, UFRGS, Porto Alegre, Brazil; d Genetics Unit, SARAH Network of Rehabilitation Hospitals, Brasilia, Brazil; e Department of Paediatric Neurology, Neurological Institute of Goiânia, Goiânia, Brazil; f Medical School, São José do Rio Preto, Brazil; g Medical Genetics Center, Fernandes Figueira Institute, FIOCRUZ, Rio de Janeiro, Brazil; h IGEIM, UNIFESP, São Paulo, Brazil; i INAGEMP, Porto Alegre, Brazil; j Dasa Genômica, São Paulo, Brazil; k Casa dos Raros, Porto Alegre, Brazil

**Keywords:** Neurologic manifestations, Natural history, Lysosomal storage diseases, Oligosaccharides

## Abstract

Alpha-mannosidosis (AM) is an ultrarare multisystemic disorder caused by alpha-mannosidase deficiency. This is the first comprehensive report on AM in Brazil, analyzing clinical and laboratory data from 14 patients diagnosed between 2001 and 2021. We summarize the patient diagnostic journey in the country, including the most common presenting symptoms, the time from disease onset to diagnosis and discuss other disease manifestations. Our findings may improve the disease awareness and understanding in the country.

## 1 Introduction

Alpha-mannosidosis (AM) is an ultrarare disease, caused by reduced enzymatic activity of the lysosomal hydrolase alpha-mannosidase (EC 3.2.1.24). The condition is inherited as an autosomal recessive trait; affected individuals have biallelic loss of function variants in the *MAN2B1* gene. It presents as a multisystemic condition with a variable disease severity and progression, reflecting the intracellular accumulation of mannose-rich oligosaccharides within the lysosomes of various tissues.

The disease has a prevalence estimated at 1:500,000 to 1:1,000,000 live births and it is predominantly characterized by facial and skeletal abnormalities, intellectual disability, hearing impairment and immune deficiency [1]. The prevalence of the disease in Brazil is unknown. However, it has been estimated a minimum birth prevalence of circa 0.2 in 1,000,000 live births [2].

The aim of this study was to survey patient demographics, clinical characteristics, and laboratory findings of Brazilian patients with AM diagnosed between 2001 and 2021.

## 2 Methods

This is a retrospective, cross-sectional study. The key inclusion criterium was a clinical and biochemical diagnosis of AM in a Brazilian patient, regardless of gender or age. There were no exclusion criteria. Clinical and biochemical data was collected through the Lysosomal Storage Diseases Brazil Network (LBN), a project approved by the Research Ethics Committee of Hospital de Clinicas de Porto Alegre (protocols #2003–0666 and #2017–0664) or direct contact and collaboration of physician assistant. Written informed consent was obtained from caregivers. The physicians were requested to review retrospective clinical data and enter them in a data collection form (see supplementary methods). The entered data consisted in available medical history, radiological, biochemical and molecular reports and the physician's clinical assessment. Patients phenotypes were classified in three categories (mild, moderate and severity) as previously proposed [3]. As needed, additional molecular or biochemical tests were offered to the healthcare providers through the LBN.

## 3 Results

**Fig. 1:**
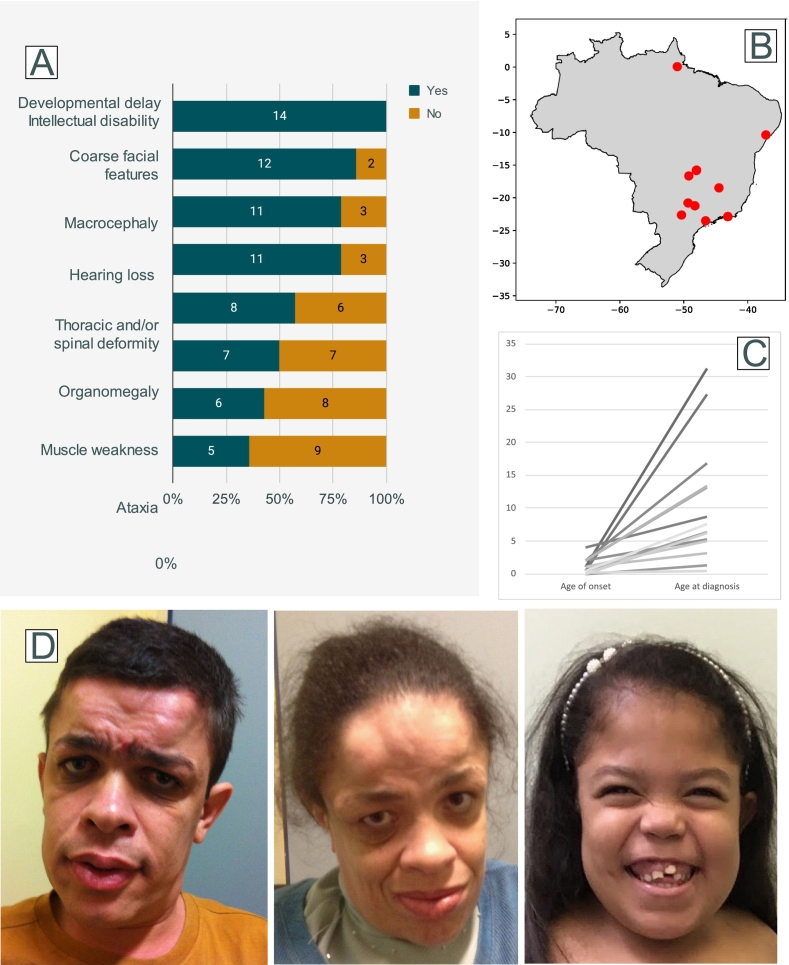
A. Most prevalent clinical manifestations of the patients. B. Birth city location of the included patients. C. Age of onset and age at diagnosis (in years) demonstrating a significant diagnostic delay. D. Facial features of subjects 1, 2 and 10. Notice frontal bossing in subjects 1 and 2 and coarse facial features in subject 10. There are some overlapping, yet distinct, characteristics as compared to other lysosomal disorders.

Fourteen patients, from 12 families were included in this study (Supplementary Table 1). The patients were from 10 different Brazilian cities (Fig. 1B). Nine of the 12 families had confirmed or suspected consanguinity. Diagnosis was confirmed by enzyme activity in all cases. None of the patients were treated with either enzyme replacement therapy or hematopoietic stem cell therapy.

The average age at the last assessment for the patients was 15.7 years. The age of onset was 1.2 years, and the diagnosis was performed by the age of 10.4 years (Fig. 1C). In all cases the diagnosis was performed either by a medical geneticist or a child neurologist.

Molecular analysis was available for five cases, with six variants reported. Two of the identified variants (p.Arg760Ter and p.Ser899Ter) were previously described nonsense variants [4,5] and were found in homozygous state in patients with a moderate phenotype. Of note, the p.Ser899Ter was recently described in 4 Brazilian patients [6]. Other four variants (p.Pro987Leu, p.Ala646Thr, p.Ser899Leu, p.Ser899=) were novel and classified as variants of unknown significance (Supplementary Table 2). Those four variants were identified in two patients that died early, either from a severe prenatal presentation (subject 6) or a fatal infectious episode (subject 13).

Data from two sibling pairs were available (subjects 1 and 2, and 3 and 4). Overall, their clinical phenotypes were highly similar. The older siblings exhibited a more severe phenotype, likely due to disease progression.

The most prevalent clinical manifestations were developmental delay/intellectual disability (14; 100 %), coarse facial features (12; 85.7 %), hearing loss (11; 78.6 %), macrocephaly (11; 78.6 %), recurrent infections (11; 78.6 %), thoracic and/or spinal deformity (8; 57.1 %), dysostosis multiplex (7; 53.8 %), organomegaly (7; 50 %), muscle weakness (6; 42.9 %), arthropathy (6; 42.9 %), and ataxia (5; 35.7 %) (Fig. 1A, D and Supplementary Table 1). Older patients had worse motor function, a history of motor regression, as well as more prominent psychiatric symptoms, in accordance with the natural history of neurological impairment in AM [7]. Additional findings included short stature, umbilical and inguinal hernia, congenital cataract, myopia, strabismus, astigmatism, and mild tricuspid and mitral valve insufficiency.

Neuroimaging studies were available for nine patients and results included diffuse thickening of the calvarium, volumetric reduction of the brain parenchyma, mild cerebellar atrophy, delayed myelination, peritrigonal and periventricular hyperintensities.

## 4 Discussion

This is the first comprehensive report about alpha-mannosidosis in Brazil. This condition is among the less common lysosomal diseases in the country, with only 7 cases diagnosed from 1982 to 2015 through LBN, as compared to 343 MPS II cases in the same period [2]. As expected for an ultrarare autosomal recessive disorder, there is a high proportion of parental consanguinity among the included families. Furthermore, there was a significant diagnostic delay with an average of 9 years after the onset of symptoms. Due to overall low awareness about this condition, patients with more severe disease may be overrepresented in this sample.

Most characteristics were consistent with findings already reported in literature [8]. All patients had either a history of developmental delay or intellectual disability. This aligns with recent data indicating a high prevalence and progressive nature of cognitive impairment in AM. [9]. In those patients with an available neuroimaging test, cognitive impairment was often, but not always, accompanied by cortical atrophy. Cerebellar atrophy was observed in older patients with ataxia.

This study has certain limitations. There was no independent review of radiological data by a skeletal dysplasia expert to assess accuracy. This may have resulted in subtle findings being overlooked, as the interpretation relied exclusively on the reports provided by the radiologists. Furthermore, no standardized neuropsychiatric testing was performed.

Besides the patients described in this study, at least another 9 patients are known in the country [10]. However, assuming a birth prevalence of 1:1,000,000, more than one hundred AM patients in Brazil are likely to remain undiagnosed. Until recently, most patients have been screened through qualitative methods, such as thin-layer chromatography of urinary oligosaccharides, which lack sensitivity and specificity for detecting AM [11]. We expect that new and more sensitive and specific biochemical tests, as well as a broader availability of molecular testing, may help diagnosing additional cases.

## 5 Conclusion

AM is probably a significantly underdiagnosed condition in Brazil. Our findings may improve the disease awareness and understanding in the country and possibly facilitate future alpha-mannosidosis diagnoses.

## CRediT authorship contribution statement

**Fabiano de Oliveira Poswar:** Writing – original draft, Methodology, Data curation. **Tamires Silva Alves:** Writing – original draft, Investigation, Data curation. **Daniel Rocha de Carvalho:** Writing – review & editing, Investigation. **Hélio van der Linden:** Writing – review & editing, Investigation. **Charles Marques Lourenço:** Writing – review & editing, Investigation. **Dafne Dain Gandelman Horovitz:** Writing – review & editing, Investigation. **Anneliese Barth:** Writing – review & editing, Investigation. **Carmen Silvia Curiati Mendes:** Data curation, Formal analysis, Investigation, Validation, Writing – review & editing. **Ana Maria Martins:** Writing – review & editing, Investigation. **Roberto Giugliani:** Writing – review & editing, Methodology, Funding acquisition, Conceptualization.

## Funding sources

This work was partially supported by an unrestricted grant from 10.13039/100007560Chiesi Farmaceutici.

## Declaration of competing interest

None.

## Data Availability

Data will be made available on request.

## Appendix A Supplementary data

Supplementary material Image 1
