# Supplementary material for: Disease profile in a cohort of Brazilian patients diagnosed with alpha-mannosidosis
Source: Mol Genet Metab Rep. 2025 Apr 11;43:101220. doi: 10.1016/j.ymgmr.2025.101220 (PMC12018040; doi:10.1016/j.ymgmr.2025.101220)
Supplement: Supplementary file 1 — Supplementary material [file mmc1.docx]

**Supplementary Material - Data Collection Form for Brazilian Cases of Alpha-Mannosidosis (translated)**

| **Patient Name:** |  | | | | |
| --- | --- | --- | --- | --- | --- |
| **Doctor:** |  | | | | |
| **Service:** |  | | | | |
| **City and State:** |  | | | | |
| **Fill in the city and state of birth of the patient and their parents** |  | | **Place of Birth** | | |
|  |  | | **City** | **State** | |
|  | **Patient** | |  |  | |
|  | **Mother** | |  |  | |
|  | **Father** | |  |  | |
| **Additional cases in the family:** | **( ) no ( ) yes – draw pedigree on the back** | | | | |
| **Consanguinity parents** | **( ) no ( ) yes – draw pedigree on the back** | | | | |
| **Medical history up to diagnosis (if you need more space, use the back)** | | | | | |
| **What motivated the diagnostic investigation? Mark all that apply and indicate the main motivator in the right-hand column.** | | | | | |
| **Sign or symptom** | | **Yes, or no, or observations** | | | **Main?** |
| MPS-like facial features | |  | | |  |
| Macrocephaly | |  | | |  |
| Ataxia | |  | | |  |
| Muscle weakness | |  | | |  |
| Intellectual disability | |  | | |  |
| Hepatosplenomegaly | |  | | |  |
| Hearing impairment | |  | | |  |
| Dysostosis multiplex | |  | | |  |
| Thoracic and/or spinal deformity | |  | | |  |
| Arthropathy | |  | | |  |
| Recurrent infections | |  | | |  |
| Affected family member | |  | | |  |
| Other(s) – specify – use the y/n verse | |  | | |  |
| Other(s) – specify – use the y/n verse | |  | | |  |
| **Diagnosis: date (even if it only has the month or year) and location** | **Day/Month/Year:** | | | | |
|  | **Laboratory that performed the diagnosis:** | | | | |
|  | **City/State of laboratory:** | | | | |
| **mannosidase dosage** | **(Material and result)** | | | | |
| **Molecular analysis** | **(Mutations found)** | | | | |
| **Other exam(s)** | **(Material, exam and result) –** use the back if necessary | | | | |
| **Medical history after diagnosis (if you need more space, use the back)** | | | | | |
| **Complementary exams** | **Use the back and/or attach** | | | | |
| **Death:** | **( ) no ( ) yes – date / /** | | | | |
| **Additional notes: use the back** | | | | | |

Supplementary Table 1 - Characteristics of individual patients.

| Id | Sex | Age of onset (years) | Main presenting manifestations | Age at diagnosis (years) | Age at last visit (years) | Diagnosing physician's specialty | Phenotype | Neurological features | Brain Imaging | Dysmorphic features and Skeletal Findings | Recurrent Infection Profile | Supportive treatment | Survival (years) | Other signs and symptoms | Enzyme activity (% of LLN) ** | Genotype |
| --- | --- | --- | --- | --- | --- | --- | --- | --- | --- | --- | --- | --- | --- | --- | --- | --- |
| 1 | F | 1 | MPS-like face, intellectual disability, arthropathy | 31.3 | 37 | Child Neurology | Moderate | Severe intellectual disability, aggressive behavior, Muscle Weakness, wheelchair user since 35 years-old | N/A | Broad forehead, depressed nasal bridge, widely spaced teeth, gingival hypertrophy, Loss of the usual sphericity of the femoral head; dorsal kyphosis; spondylodysostosis; sinovitis, osteopenia | Multiple recurrent infections | Fluoxetine, topiramate for the management of behavioral issues | alive | Hearing loss, Dysphagia, Hyperprolactinemia | 9.1 (P) | N/A |
| 2 | M | 0.5 | MPS-like facies, macrocephalus, developmental delay | 27.3 | 30 | Child Neurology | Moderate | Severe intellectual disability, Muscle Weakness | Head CT: Volumetric reduction of the brain parenchyma. Diffuse thickening of the cranial vault. | Broad forehead, depressed nasal bridge, widely spaced teeth, gingival hypertrophy, Degenerative spondylodiscopathy; degenerative arthropathy, joint effusion | Multiple recurrent infections | Fluoxetine | alive | Hearing loss | 0.3 (L) | N/A |
| 3 | F | 4 | Hearing loss, affected sibling | 8.7 | 12.6 | Medical Genetics | Moderate | Language delay, Moderate Intellectual disability | N/A | Coarse Facies, Macrocephaly, dolicocephaly, broad forehead, Dysostosis multiplex, Arthropathy, expansion of the distal femoral metaphyses, expansion of the tibial diaphyses | Pharyngitis and otitis media | N/A | alive | astigmatism, hyperopia, vacuolated lymphocytes, allergic rhinitis, trace mitral insufficiency | 1.7 (L) | c.2278C>T  (p.Arg760*) homozygous |
| 4 | F | 2 | Hearing loss, intellectual disability, affected sibling | 16.8 | 21.8 | Medical Genetics | Moderate | Moderate Intellectual disability; apathy | normal | Coarse Facies, Macrocephaly, Dysostosis multiplex, Calvarial thickening, widening of the ribs at T8–T12, expansion of the distal ulnar and femoral metaphyses, thoracic kyphosis | Pharyngitis and otitis media | N/A | alive | vacuolated lymphocytes, hyperopia | 1.0 (L) | c.2278C>T  (p.Arg760*) homozygous |
| 5 | F | 2 | Developmental delay, hearing loss, hepatosplenomegaly | 5.3 | 14.9 | Medical Genetics | Moderate | Developmental delay, Ataxia and pyramidal findings, wheelchair user since 14 years old, epilepsy | normal | Coarse Facies, Calvarial thickening, kyphosis, arthropathy, acetabular irregularities | N/A | carbamazepine due to epilepsy | alive | epilepsy | 2.2 (L) | N/A |
| 6 | M | prenatal | Hydrops featalis, developmental delay and recurrent infections | 1.3 | 2.8 | Medical Genetics | Severe | Global develomental delay, Ataxia, hypotonia | T2 hyperintensity in the peritrigonal region | Coarse Facies, Macrocephaly | Recurrent otitis media | N/A | 2.8 | Organomegaly, Hearing loss, Failure to Thrive | 73.3 (DBS) | c.2960C>T (p.Pro987Leu); c.1936G>A (p.Ala646Thr) |
| 7 | M | 0.5 | Hypotonia and global developmental delay | 6.3 | 8.1 | Medical Genetics | Moderate | GDD/Intellectual Disability, Behavior disorder, Ataxia, hypotonia | Mild cerebelar atrophy | Coarse Facies, Macrocephaly | N/A | N/A | alive | Organomegaly, Strabism, Myopia, Abnormal transferrin isoelectric focusing | 23.3 (DBS) | N/A |
| 8 | M | 2 | MPS-like face, developmental delay, and hearing loss | 13.1 | 23.8 | Medical Genetics | Moderate | Moderate Intellectual Disability, Ataxia, Muscle Weakness | normal head CT at 12 years-old | Coarse Facies, Macrocephaly, Macroglossia, Dysostosis multiplex, platispondyly | N/A | N/A | alive | Hearing loss | 3.3 (L) | N/A |
| 9 | M | 2 | Developmental delay, hepatosplenomegaly, MPS-like facies, macrocephaly | 13.4 | 21.8 | Medical Genetics | Moderate | Speech delay, Intellectual Disability, Hemiparesis, muscle weakness, lower limb spasticity, bilateral Babinski sign, ataxia | Global cortical volumetric reduction, hyperintense signal on FLAIR and T2 in the periventricular white matter adjacent to the frontal and occipital horns of the lateral ventricles Cerebellar volumetric reduction and thinning of the corpus callosum. | Coarse Facies, Macrocephaly, Dysostosis multiplex, Arthropathy | Recurrent otitis media until 4-years-old | N/A | alive | Hearing loss, Congenital cataracts, strabism, optic nerve pallor | N/D (P) | c.2696C>A  (p.Ser899*) homozygous |
| 10 | M | 1 | Hearing loss, developmental delay, hepatosplenomegaly | 3.2 | 5.6 | Medical Genetics | Mild | Mild intellectual disability, Normal | N/A | Relative macrocephaly | Recurrent infections | N/A | alive | Bilateral inguinal hernia | 0.3 (L) | N/A |
| 11 | M | 1 | Recurrent infections, developmental delay and hypotonia | 5 | 8.9 | Medical Genetics | Mild | Intellectual Disability, Ataxia, Muscle Weakness | Brain MRI: cerebelar atrophy | Mild coarse Facies | Recurrent infections | N/A | alive | N/A | 30.0 (DBS) | N/A |
| 12 | F | prenatal | Increased nuchal transluscence, MPS-like facies, macrocephaly, hearing impairment | 6.2 | 9.6 | Medical Genetics | Moderate | Intellectual Disability | Brain MRI at 6 years: T2/Flair hyperintense signal in periventricular white matter, corona radiata and semi-oval centers. Dilation of perivascular spaces. | Depressed nasal bridge, prognatism, midface retrusion, gingival hypertrophy, macrocephaly, Dysostosis multiplex, leg length discrepancy, scoliosis, delayed ossification of the odontoid process, calvarial thickening, Arthropathy | Upper airway infections, pneumonia | N/A | alive | Organomegaly, umbilical hernia; myopia | 0.3 (L) | N/A |
| 13 | F | 0.2 | Recurrent infections, developmental delay and hypotonia | 0.5 | 1.3 | Medical Genetics | Moderate | Developmental delay, Muscle Weakness | Brain MRI: myelination delay | N/A | Pneumonias, including Covid-19, complicating with fatal cardiopulmonary arrest. | N/A | 1.3 | Organomegaly, Hearing loss, Macrocephaly | 40.0 (DBS) | c.2696C>T (p.Ser899Leu); c.2697G>A (silent) |
| 14 | M | 0.5 | Recurrent infections, developmental delay, Hearing loss, spinal deformity | 7.6 | 22 | Medical Genetics | Moderate | Moderate Intellectual Disability, Unable to walk long distances | Brain MRI: brain atrophy | Coarse Facies, gingival hypertrophy, small spaced teeth, Dysostosis multiplex, pectus carinatum, short stature | Recurrent upper airway infections | N/A | alive | Mild mitral and tricuspid insufficiency | 0.2 (L) | N/A |

F, female; GDD, global developmental delay; M, male; N/A, not available; N/D, not detected; LLN, lower limit of normality; UNK, unknown. *Possible parental consanguinity: family denied consanguinity, however both parents were born in a small town (DATASUS population 2012: 13,992). ** Enzyme activity was measured by different methods in either plasma (P), leukocytes (L) or dried blood spots (DBS).

Supplementary Table 2 – Variants described in this study

| c.DNA (NM_000528.4) | Protein (NP_000519.2) | ACMG / Clingen criteria | Classification | References (PMID) |
| --- | --- | --- | --- | --- |
| c.2278C>T | p.Arg760Ter | PVS1_verystrong; PM2_supporting; PM3_verystrong | Pathogenic | 9758606 |
| c.2960C>T | p.Pro987Leu | PM2_supporting | VUS | Not reported |
| c.1936G>A | p.Ala646Thr | PM2_supporting | VUS | Not reported |
| c.2696C>A | p.Ser899Ter | PVS1_verystrong; PM2_moderate; PP5_supporting | Pathogenic | 9915946 |
| c.2696C>T | p.Ser899Leu | PM2_supporting | VUS | Not reported |
| c.2697G>A | p.Ser899= | PM2_supporting | VUS | Not reported |
